# Factors affecting the yield of microRNAs from laser microdissectates of formalin-fixed tissue sections

**DOI:** 10.1186/1756-0500-5-40

**Published:** 2012-01-19

**Authors:** Santosh Kumar Patnaik, Eric Kannisto, Sai Yendamuri

**Affiliations:** 1 Department of Thoracic Surgery, Roswell Park Cancer Institute, Elm and Carlton Streets, Buffalo, NY 14203, USA

**Keywords:** Cresyl violet, Formalin-fixed tissue, Hematoxylin-eosin, Laser microdissection, MicroRNA, RNA isolation

## Abstract

**Background:**

Quantification of microRNAs in specific cell populations microdissected from tissues can be used to define their biological roles, and to develop and deploy biomarker assays. In this study, a number of variables were examined for their effect on the yield of microRNAs in samples obtained from formalin-fixed paraffin-embedded tissues by laser microdissection.

**Results:**

MicroRNA yield was improved by using cresyl violet instead of hematoxylin-eosin to stain tissue sections in preparation for microdissection, silicon carbide instead of glass fiber as matrix in RNA-binding columns, and overnight digestion of dissected samples with proteinase K. Storage of slides carrying stained tissue sections at room temperature for up to a week before microdissection, and storage of the microdissectates at room temperature for up to a day before RNA extraction did not adversely affect microRNA yield.

**Conclusions:**

These observations should be of value for the efficient isolation of microRNAs from microdissected formalin-fixed tissues with a flexible workflow.

## Background

Laser microdissection (LMD) [1] is commonly used for the selective isolation of cell populations from tissues for molecular analyses. LMD is performed under microscopy, and cells are dissected out using a laser beam after they are identified by features such as histologic morphology. Quantification of the ultrashort, non-coding microRNAs in microdissected cells is an effective approach to understand the physiological roles of microRNAs [2-5] as well as to characterize microRNA dysregulation in diseases [6-10]. Unlike the much longer transcript mRNAs, microRNAs are resistant to fragmentation, and this permits the use of archived tissue material like formalin-fixed and paraffin-embedded (FFPE) specimens instead of fresh-frozen ones for reliable microRNA measurements for various studies [11-13]. Many of the variables that affect the recovery of microRNAs from macroscopic FFPE tissues have been identified [14-18]. However, the amount of cellular material obtained with LMD is minute, and the technique itself introduces conditions such as the presence of histologic dyes in the dissectates. In this study, we have examined some such factors of practical importance that can affect the yield and quality of microRNAs from LMD microdissectates of FFPE tissues for downstream analysis. One of the main advantages of developing biomarkers using microRNAs is the ability to use FFPE specimens. Therefore, our study focused completely on the use of FFPE specimens and no comparison to fresh frozen tissue was attempted.

## Results and discussion

We obtained FFPE tissues of human lung cancers or their xenografts grown in mice for this work. Tissues were cut into 8 μm-thick sections, which were then placed on glass slides covered with polyethylene naphthalate (PEN) membrane. The sections were deparaffinized and stained with either hematoxylin and eosin (H&E), or cresyl violet (CV), and used for LMD within a day with a pulsed ultraviolet laser on a Leica^® ^LMD6000 system. For some experiments, areas of tissue sections were dissected out along with PEN membrane by hand using a surgical blade. To obtain replicate samples, morphologically identical quadrants of stained serial sections were cut. Dissectates were lysed with proteinase K and total RNA was extracted by affinity chromatography using the Ambion^® ^RecoverAll™ Total Nucleic Acid Isolation, or Norgen Biotek^® ^FFPE RNA Purification kits that respectively use silica or glass fiber (GF), or carborundum or silicon carbide (SiC) as the RNA-binding matrix. Total RNA, with microRNA in an amount expected to be a constant proportion of that of total RNA, was eluted from columns using identical volumes of water, and quantified using RiboGreen dye in a fluorescence assay [19], or by measuring absorbance at 260 nm. Identical volumes of different RNA preparations were used for Applied Biosystems^® ^TaqMan™ microRNA assays, based on reverse transcription-PCR (RT-PCR) [20], for microRNA *miR-16*, an abundant and ubiquitous microRNA (e.g., [21]), and *RNU6-2 *(*U6B*), a 45 base-long, housekeeping nucleolar RNA. Inter-group differences were analyzed using t tests assuming equal variances. *P *values determined in different statistical tests were two-tailed and a cut-off of 0.05 was used to appraise significance.

**Figure 1:**
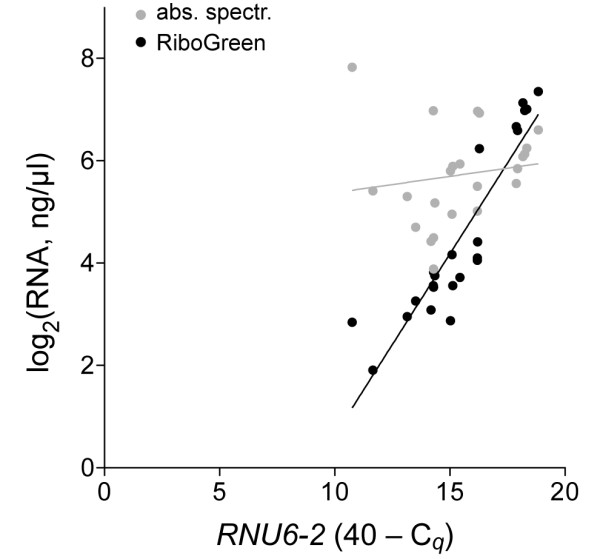
**Scatter-plots of RNA concentration and *RNU6-2 *measurements of RNA from dissectates of formalin-fixed tissue sections**. Total RNA in 23 samples was quantified by RiboGreen assay (*black*) or absorbance spectrophotometry at 260 nm (*grey*). Level of *RNU6-2 *in the RNA preparations was determined as quantification cycle (*C_q_*) values obtained in reverse transcription-PCR assays. The best lines of fit with the least squares method are also shown.

**Figure 2:**
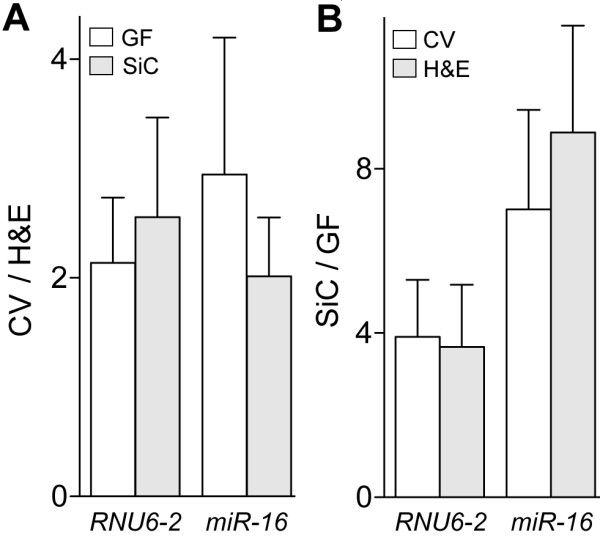
**Effect of histologic stain and RNA-binding matrix in spin-columns on RNA yield**. Yields with cresyl violet (*CV) *stain relative to hematoxylin and eosin (*H&E*) for glass fiber (*GF*) and silicon carbide (SiC) columns (*A*), and with SiC relative to GF columns for both stains (*B*) are plotted as means with their standard errors for dissectates from three tissues. Log_2_-transformed *RNU6-2 *and *miR-16 *levels were determined from C*_q _*values obtained in reverse transcription-PCR assays.

An analysis of RNA preparations from 23 different dissectates from xenografts showed that *RNU6-2 *levels correlated well with total RNA estimations by RiboGreen assay with a Pearson coefficient of 0.91 (95% confidence interval = 0.79-0.96; *P *< 0.01) whereas there was no significant correlation with total RNA quantifications by absorbance at 260 nm (*P *= 0.15; Figure 1). RiboGreen assay was thus deemed as more precise than absorbance spectrophotometry for RNA samples of low concentration, as has been observed by others [22], and was used to assess total RNA for the rest of the study. H&E and CV are nucleic acid-binding stains that can possibly interfere with RNA extraction, and their use can differentially affect RNA degradation during the processing steps of staining [23-27]. While others have shown that the use of stains other than H&E and CV influences RNA yield from LMD samples, recent reports have suggested superiority of the use of CV over H&E to obtain RNA for downstream gene expression profiling. Therefore, we decided to test CV and H&E stains in this study. We compared small RNA yields from H&E- or CV-stained replicate dissectates from three xenografts by measuring *RNU6-2 *and *miR-16 *levels. In RNA extracted using GF columns, *RNU6-2 *and *miR-16 *levels respectively were an average of 2.1 and 3.0 times higher with CV than H&E (Figure 2A). With SiC columns too, *RNU6-2 *and *miR-16 *levels respectively were on average 2.6 and 2.0 times higher with CV than H&E. In paired t tests disregarding the column-type, the improvements in *RNU6-2 *and *miR-16 *yields were significant (*P *values of 0.02 and 0.01, respectively).

The efficacies of the two types of RNA-binding columns that used wither GF or SiC as the RNA-binding matrix were also compared. For this, proteinase K lysates were prepared from dissectates from three xenografts and divided into two equal portions, each of which was used for the two types of columns. As shown in Figure 2B, with CV-stained dissectates, *RNU6-2 *and *miR-16 *levels respectively were an average of 3.9 and 7.0 times higher with SiC columns than GF columns. When H&E was the stain, *RNU6-2 *and *miR-16 *levels respectively were on average 3.7 and 7.9 times higher with SiC columns than GF columns. These improvements in *RNU6-2 *and *miR-16 *yields, significant in paired t tests disregarding the histologic stain (both *P *values < 0.01), could be because of differences in column design and not necessarily because of a better efficacy of the SiC matrix per se. Because of convenience during the staining step and with assessment of histological morphology, we decided to use H&E stain for the rest of the experiments of this study. As the goal of the experiments was to assess improvements in yield, the influence of downstream variables such as those during poteinase K digestion or storage of the microdissectates was expected to not deny superiority of CV stain over H&E.

**Figure 3:**
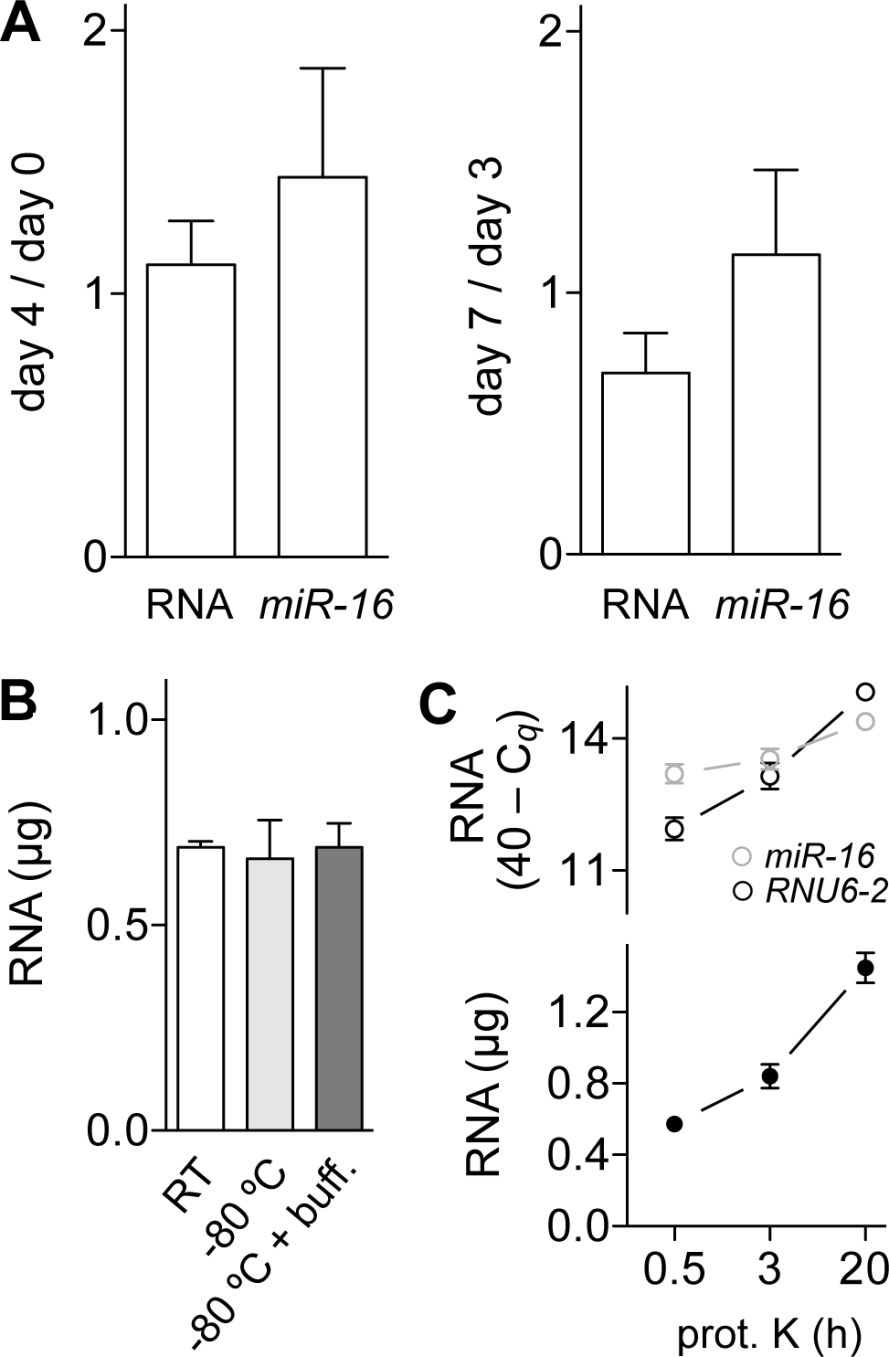
**Effect of age of slides and dissectates, and proteinase K treatment duration on RNA yield**. *A*. Total RNA and *miR-16 *yields from laser microdissectates from three tissues prepared from four or seven day-old slides relative to zero or three day-old ones, respectively. *B*. Total RNA yield from identical laser dissectates from zero day-old slides stored in duplicate at room temperature (*RT*), or at -80°C with or without buffer (*buff*.) for a day. *C*. Total RNA yield (*filled circles*) and levels of *RNU6-2 *(*black empty circles*) and *miR-16 *(*grey empty circles*) from identical dissectates treated in triplicate with proteinase K (*prot. K*) for 0.5, 3 or 20 h. Means and their standard errors are plotted. Log_2_-transformed *RNU6-2 *and *miR-16 *levels were determined from quantification cycle (C*_q_*) values obtained in reverse transcription-PCR assays. Total RNA was quantified by RiboGreen assay. Hematoxylin and eosin was used as the histologic stain, and silicon carbide columns were used for RNA isolation.

To test effect on RNA yield of duration of storage of stained slides at room temperature under ambient conditions before dissection and RNA extraction, replicate sections from three xenografts were used for dissection on the same day (day 0) the slides were prepared or after a period of 3-7 days. RiboGreen and *miR-16 *assays of the RNA preparations showed that RNA yields were not reduced at day 4 compared to day 0, or at day 7 compared to day 3 (Figure 3A). This observation indicates that slides can be prepared and stored for at least a week before LMD is performed without an adverse effect on microRNA yield. The effect of different storage conditions for dissectates before RNA extraction was also examined (Figure 3B). There was no significant difference in RNA yield as measured by RiboGreen assay between LMD samples kept at room temperature for a day in a dry state, or at -80°C either in a dry state or in the tissue lysis buffer provided with the RNA extraction kit.

As expected from previous studies on RNA extraction from FFPE tissues (e.g., [16]), RNA yield improved significantly when the duration of proteinase K treatment was extended (Figure 3C). RiboGreen, *RNU6-2 *and *miR-16 *measurements respectively were on average 1.5, 2.3 and 1.3 times higher when the duration was increased to 3 h at 55°C from 15 min at 55°C followed by 15 min at 80°C (*P *values of 0.02, 0.04 and 0.36, respectively). Extending treatment time from 3 h to 20 h resulted in 1.7, 3.8 and 1.8 times higher RiboGreen, *RNU6-2 *and *miR-16 *measurements, respectively (*P *values of < 0.01, < 0.01 and 0.03, respectively).

**Figure 4:**
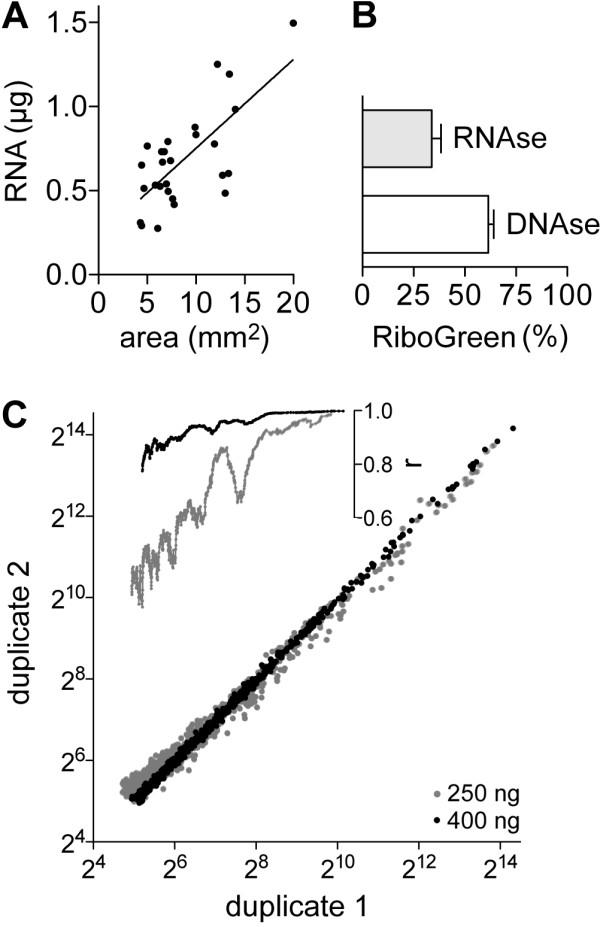
**Assessment of RNA prepared from FFPE tissue microdissectates**. *A*. Scatter-plot of area and RNA yield as per RiboGreen assay for 27 tissue samples obtained by laser microdissection (LMD). The best line of fit with the least squares method is shown. *B*. Measurements in RiboGreen assay following treatment of four RNA preparations with RNAse A or DNAse I enzyme relative to treatment without either. Means with their standard errors are shown. *C*. Microarray signal values (*dots*) and inter-duplicate Pearson correlation coefficient, r (*lines*) for 747 microRNAs measured in duplicate using 250 (*grey*) or 400 ng (*black*) of RNA prepared from an LMD sample. A rolling window of width 99 along the × axis was used for calculating value of r at the mid-window abscissa.

To assess the relation of dissectate quantity and RNA yield, epithelial components of 27 human non-small cell lung cancers were isolated by LMD from H&E-stained FFPE tissue sections, and digested with proteinase K at 55°C overnight. RNA from the lysates was prepared using the kit from Norgen Biotek^®^. As shown in Figure 4A, there was a significant Pearson correlation (r = 0.71, 95% confidence interval = 0.45-0.86) between cross-sectional areas of dissectates and RiboGreen measurements of RNA prepared from them, with an average of 84 ng RNA obtained per mm^2 ^area. The average RNA yields from the 17 tumors of adenocarcinoma histology (78 ng/mm^2^) and from the 10 tumors of squamous cell carcinoma histology (92 ng/mm^2^) were not statistically different from each other (*P *= 0.24). RiboGreen assay of four different RNA preparations that were treated with DNAse I, RNAse A or neither at 37°C for 1 h showed that 37%-39% of the nucleic acids in the RNA preparations was DNA and not RNA (Figure 4B). To assess the suitability of the RNA for microRNA quantification using microarrays, 250 or 400 ng of one RNA sample was labeled with Hy3™ dye and hybridized in duplicate to Exiqon^® ^miRCURY™ locked nucleic acid (LNA) microarrays. With both 250 and 400 ng input, about 56% of the 1291 microRNAs detectable by the microarrays were identified as expressed. However, microarray signals were stronger with higher RNA input (Figure 4C). E.g., 21% of expressed microRNAs had signal values of > 200 with 400 ng RNA whereas the value was 17% for 250 ng. Inter-duplicate correlation analyses showed that microarray signals were likely more accurate and less noisy when more RNA was used (Figure 4C). Comparison of microarray signal from RNA prepared from microdissectates with that from a commercially available human 'universal reference' RNA, which was used for the reference channel of the two-color microarrays, showed that the microRNA isolation method did not adversely affect RNA labeling and hybridization for microarray analysis (see Additional file 1: Figure S1).

## Conclusions

To summarize, this study suggests that microRNA yields from LMD samples obtained from FFPE tissues can be improved by using CV instead of H&E as histologic stain, SiC instead of GF as matrix in RNA-binding columns, and overnight digestion with proteinase K. Storage of stained slides at room temperature for up to a week before LMD, and storage of LMD samples at room temperature for up to a day before RNA extraction does not seem to adversely affect microRNA yield. RNA prepared as per the methods used in this study, though containing DNA as well, appear to be suitable for microRNA quantification by RT-PCR or microarray hybridization. These observations should allow for efficient isolation of microRNAs from microdissectates prepared from FFPE tissues with a more manageable and flexible workflow.

## Methods

### Ethics statement

The research presented here was approved under protocol ID I129008 by the Institutional Review Board of the Roswell Park Cancer Institute (RPCI). Informed consent specifically for this study was not obtained from the participants as such a requirement was waived under the protocol.

### Tissues and microdissection

FFPE tissues of human non-small cell lung cancer and their xenografts in immunodeficient mice were kindly provided by, respectively, the core pathology facility of RPCI, and Dr. Bonnie Hylander of the Department of Immunology, RPCI. Tissue blocks were cut on a CUT4055 rotary microtome (Triangle Biomedical Sciences^®^, Durham, NC) into 8 μm-thick sections, which were placed on glass slides covered with a PEN membrane (Leica^®^, Wetzlar, Germany). Slides were dried overnight, de-paraffinized with xylene and rehydrated using a graded ethanol series (100%, 99%, 75%, and 50%, by volume in water) for staining with either CV (5 mg/ml in 20% ethanol and 1.5% acetic acid at pH 2.5; Ambion^®^, Austin, TX), or H&E using Harris hematoxylin (Polysciences^®^, Warrington, PA) followed by eosin Y (5 mg/ml; Fisher Scientific^®^, Pittsburgh, PA) according to protocols provided by the manufacturers. Slides were then dehydrated using a reverse graded ethanol series and xylene, and used for LMD within a day. LMD was performed with a pulsed ultraviolet laser on an LMD6000 system (Leica^®^) at 50×-200× magnification with laser power, speed and specimen-balance settings of 98, 2 and 11, respectively, in a room with > 35% humidity. Dissectates were collected in 0.5 ml polypropylene tubes. The duration of LMD to obtain a dissectate sample varied from 15 to 120 min. Dissectates were also obtained by manually excising tissue sections along with the PEN membrane with a scalpel blade. Morphologically identical quadrants of serial sections were cut for replicate samples. All work was done with precautions to maintain an RNAse-free environment.

### Isolation of RNA

Total RNA was isolated using protocols and reagents supplied with the RecoverAll™ Total Nucleic Acid Isolation (product number AM1975; Ambion^®^), miRCURY™ Cell and Plant Tissue RNA Isolation (product number 300110; Exiqon^®^, Vedbaek, Denmark), and FFPE RNA Purification (product number 25300; Norgen Biotek^®^, Thorold, Canada) kits. All three kits contain spin columns with an RNA-binding matrix: ~0.01 g silica in case of RecoverAll™, and ~0.1 g SiC powder in the other two. The columns provided with the kits of Exiqon^® ^and Norgen Biotek^® ^are identical as Exiqon^® ^procures the columns from Norgen Biotek^®^. Lysis of tissues and treatment with proteinase K at 55°C before a lysate was loaded on columns were done using reagents and instructions provided with the FFPE RNA Purification or the High Pure™ miRNA Isolation (product number 05 080 576 001; Roche^®^, Indianapolis, IN) kits. The concentration of proteinase K in the reactions set up as per the methods recommended for the two kits were 0.65 and 5.7 μg/μl respectively. Loading of lysates on a column and column washes were done using solutions and protocols supplied with the kit for that column. RNA was eluted from a column using either 50 or 100 μl water with the same volume used for all elutions in any given experiment.

### Semi-quantification of RNAs by RT-PCR

TaqMan™ MicroRNA RT-PCR assay (Applied Biosystems^®^, Foster City, CA), with identification number 391, was used to measure microRNA *miR-16*. A similar assay was designed as per principles outlined in previous studies [20,28], validated (see Additional file 2: Figure S2), and used to quantify the small nucleolar RNA *RNU6-2 *(also known as *U6B*). Sequences (and final concentrations in reactions) of the RT, and forward and reverse PCR primers, and the TaqMan™ probe were, respectively, GTCGTA TCCAGT GCAGGG TCCGAG GTATTC GCACTG GATACG ACAAAA ATAT (50 nM), GTGCAG GGTCCG AGGT (1 μM), GCAAGG ATGACA CGCAAA T (1 μM) and TATGGA ACGCTT CACGA (200 nM). For the RT-PCR assays, 5 μl each of RNA preparations were reverse transcribed using RNA-specific primers and reagents provided with the TaqMan™ MicroRNA Reverse Transcription Kit (Applied Biosystems^®^). RT reactions were used as templates in 40 cycle-PCR reactions on a 7900HT real-time PCR machine (Applied Biosystems^®^). Quantification cycle (C*_q_*) values, approximately inversely proportional to log_2 _values of analyte RNA concentrations, were obtained with SDS™ software (version 2.4; Applied Biosystems^®^). The average of C*_q _*values of triplicate PCR reactions was used for analysis. C*_q _*values were > 40 for negative controls, for which RT reactions were performed without RNA. C*_q _*values were subtracted from 40 to obtain measurements directly proportional to log_2 _values of analyte RNA concentrations.

### RNA quantification using RiboGreen assay

Nucleic acid concentration in RNA preparations was quantified in duplicate with Quant-it™ RiboGreen RNA reagent (Invitrogen^®^) as per the method suggested by the manufacturer. Yeast tRNA (Ambion^®^) was used to prepare standards of known RNA concentration. RNA samples (1-4 μl) were diluted to 100 μl using 10 mM tris hydrochloride with 1 mM ethylenediaminetetraacetic acid at pH 7.5 (CellGro^®^, Manassas, VA), and mixed with 100 μl of the buffer with 200- or 2000-fold diluted RiboGreen (for high- and low-range assays, respectively). Fluorescence at 535 nm following excitation at 485 nm was measured for 0.1 s on a Victor Wallac™ 1420 plate reader (Perkin Elmer^®^, Waltham, MA). Unknown RNA concentrations were extrapolated from standard curves generated for yeast tRNA.

### Nuclease treatment of RNA preparations

Bovine pancreas RNAse A (DNAse- and proteinase-free) and recombinant DNAse I (RNAse-free) were obtained from Fermentas^® ^(Glen Burnie, MD). Ten μl of nuclease reactions were set up at 37°C for 1 h using 1 U of either enzyme, buffer provided by Fermentas^® ^for use with DNAse I, and 8 μl of RNA preparation containing < 0.1 μg RNA as per RiboGreen assay. Control reactions using yeast tRNA (0.1-0.2 μg) confirmed completeness of the RNAse reactions and absence of RNAse activity in the DNAse I stock.

### MicroRNA profiling using LNA microarrays

This work was performed as a commercial service by Exiqon^® ^(Vedbaek, Denmark) using their 6th generation miRCURY™ LNA™ microarrays. Each array had more than 2383 LNA capture probes for multiple RNAs of human, mouse, rat, and some viruses printed in quadruplicate on randomly distributed spots of 100 μm diameter with an inter-spot distance of 210 μm. A total of 1304 probes targeted 1291 human microRNAs, including 66 proprietary ones (miRPlus™, Exiqon^®^), and 23 non-microRNA human small RNAs with < 200 nucleotides, including the *5S *ribosomal RNA and the *RNU6*-*2 *small nucleolar RNA (*U6B*). Every microRNA was recognized by only one of the 1276 probes for microRNAs. Eight probes recognized two microRNAs each, and three and six microRNAs were recognized by one probe each. For simplicity, the signals from such probes were considered as representing single microRNAs. Before hybridization to a microarray, 0.25 or 0.4 μg of an RNA sample, reduced in volume at room temperature in a speed-vacuum apparatus, and a human 'universal reference' total RNA preparation made by mixing the RNA pools provided in the FirstChoice^® ^Human Total RNA Survey Panel (product number AM6000, Ambion^®^, Austin, TX) were 3'- or 5'-end-labeled with Cy3-like Hy3™ or Cy5-like Hy5™ (Exiqon^®^) dyes, respectively, using miRCURY™ LNA™ microRNA Hi-Power Labeling kits (Exiqon^®^). Microarrays were scanned for analysis using ImaGene^® ^software (version 9; BioDiscovery^®^, Los Angeles, CA). Examinations of the scans and analyses of microarray signal values for 52 spiked-in synthetic, small RNAs showed that all labeling reactions and hybridizations were of good quality. Hy3™ and Hy5™ signal values were processed with the limma [29] Bioconductor package (version 3.6.9) for R (version 2.12). Correction for background noise was done using the normexp method [30] with an 'offset' value of 10, and was followed by within-array normalization using the global loess regression method with a 'span' value of 1/3 [31]. Microarray signal values were then identified as summarized Hy3™ values which were the means of values from the quadruplicate probe-spots when the maximum was < 1.5 times the minimum, or the medians if otherwise. MicroRNAs recognized by probes for which the microarray signal values were > 3 times the summarized microarray signal value for probe-less empty microarray spots (1108 total) were considered as expressed.

### Other

Unless specified otherwise, statistical analyses and graphical plotting were done in Prism™ software (version 5.0 d; GraphPad Software^®^, La Jolla, CA), *P *value of 0.05 was the cut-off for deciding significance, and t tests were two-tailed, assumed equal variances, and used paired samples when possible.

## Availability of supporting data

Both raw and processed microarray data can be obtained from the Gene Expression Omnibus repository of the National Center for Biotechnology Information, USA, with accession number GSE31946.

## Competing interests

The authors declare that they have no competing interests.

## Authors' contributions

All authors contributed to the conception and design of the study, and analyzed data. EK performed the experiments. SKP wrote the manuscript. All authors read and approved the final manuscript.

## Supplementary Material

Additional file 1 **Figure S1**. Labeling of RNA prepared from dissectates and hybridization to microarrays. Two-hundred-fifty or 400 ng each of a human 'universal reference' total RNA (Ambion^®^) were labeled with Hy5™ dye and the same amounts of RNA prepared from laser microdissected tissue using FFPE RNA Purification kit (Norgen Biotek^®^) were labeled with the Hy3™ dye, and co-hybridized to a locked nucleic acid microarray (Exiqon^®^). Fifty-two different synthetic artificial microRNAs were exogenously added to the RNAs before labeling. Scatter-plots of the Hy5™ and Hy3™ microarray signal values for the 52 spike-ins, and their linear regression lines (ordinary least squares method) are shown. The slopes of the lines are 0.70 and 0.81 for 250 and 400 ng RNA input, respectively, suggesting that the method used to isolate RNA from dissectates did not negatively affect the labeling and hybridization of the RNA. Click here for file

Additional file 2 **Figure S2**. Validation of a custom reverse transcription (RT)-PCR assay for RNU6-2. A. Quantification cycle (C*_q_*) values were determined for 40, 15 or 5 ng total RNA isolated from cells derived from the A549 human lung cancer cell-line. The TaqMan™ microRNA RT-PCR assay with ID 1093 from Applied Biosystems^® ^(*ABI*) or a similar but custom assay for the *RNU6-2 *nucleolar RNA were used. The two assays were different for only the primers and probes. The linear regression line (ordinary least squares method) for the scatter-plot is also shown. The Pearson correlation coefficient is > 0.99 (*P *= 0.02). *B*. An ethidium bromide-stained agarose gel (2%) after electrophoresis of the RT-PCR products for the assays with 40 ng RNA input was transilluminated with ultraviolet light and photographed. Sizes of DNA molecular weight markers (Invitrogen^®^, Carlsbad, CA) in base-pairs (*bp*) are shown. The RT-PCR product expected in the custom assay has a size of 75 bp. Click here for file
