# Supplementary figures and images for: Factors affecting the yield of microRNAs from laser microdissectates of formalin-fixed tissue sections
Source: BMC Res Notes. 2012 Jan 19;5:40. doi: 10.1186/1756-0500-5-40 (PMC3284398; doi:10.1186/1756-0500-5-40)

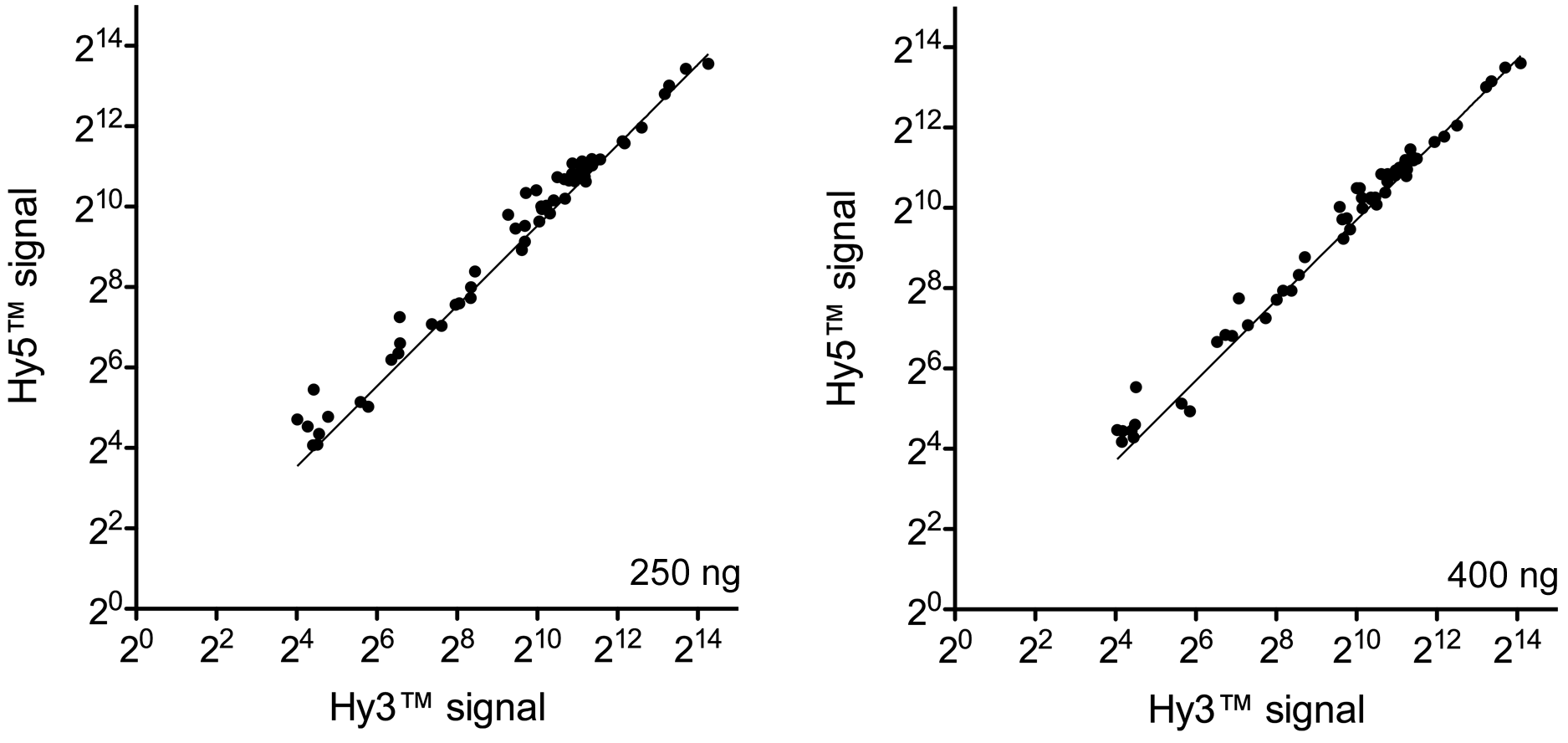

Supplement: Additional file 1 — Figure S1. Labeling of RNA prepared from dissectates and hybridization to microarrays. Two-hundred-fifty or 400 ng each of a human 'universal reference' total RNA (Ambion®) were labeled with Hy5™ dye and the same amounts of RNA prepared from laser microdissected tissue using FFPE RNA Purification kit (Norgen Biotek®) were labeled with the Hy3™ dye, and co-hybridized to a locked nucleic acid microarray (Exiqon®). Fifty-two different synthetic artificial microRNAs were exogenously added to the RNAs before labeling. Scatter-plots of the Hy5™ and Hy3™ microarray signal values for the 52 spike-ins, and their linear regression lines (ordinary least squares method) are shown. The slopes of the lines are 0.70 and 0.81 for 250 and 400 ng RNA input, respectively, suggesting that the method used to isolate RNA from dissectates did not negatively affect the labeling and hybridization of the RNA. [file 1756-0500-5-40-S1.TIFF]

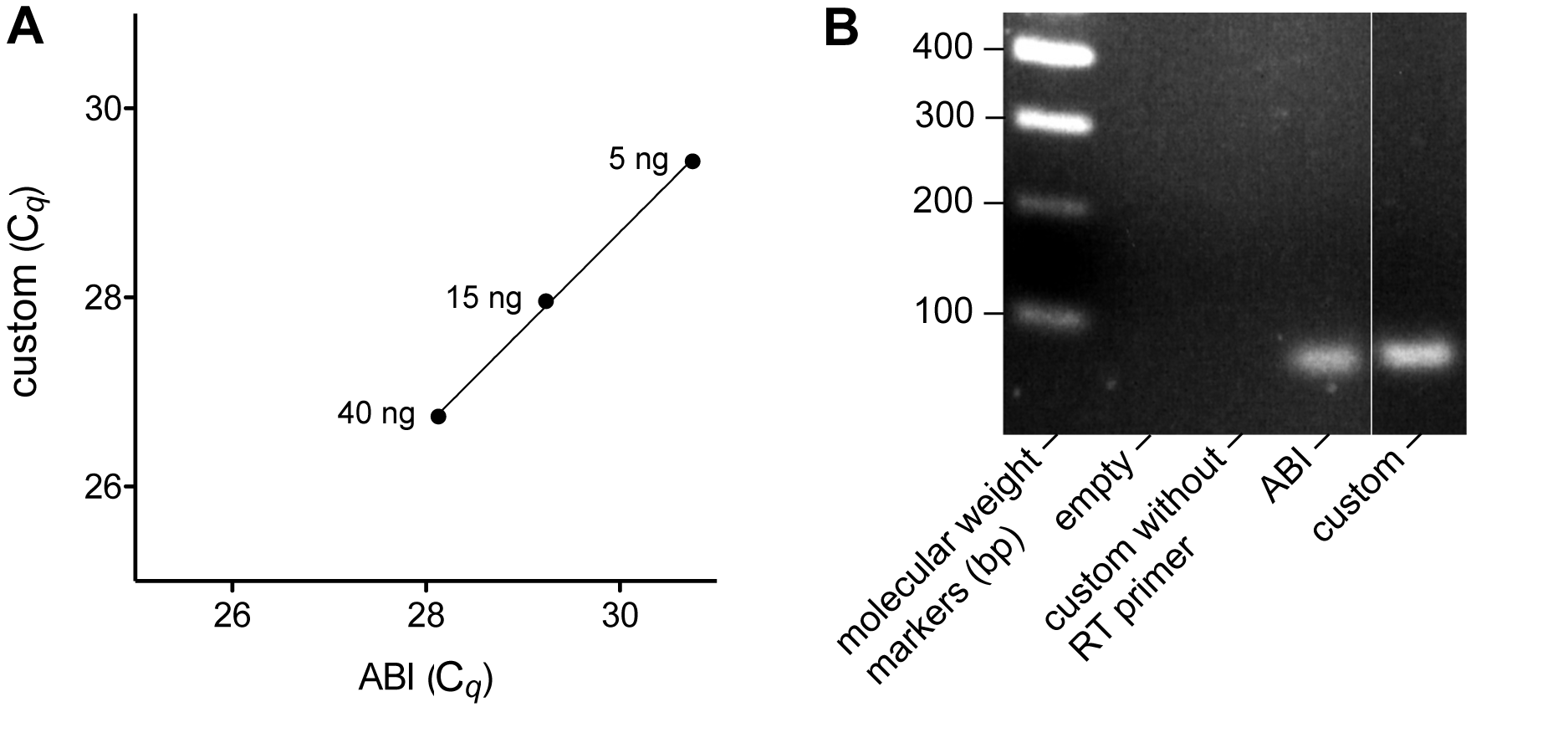

Supplement: Additional file 2 — Figure S2. Validation of a custom reverse transcription (RT)-PCR assay for RNU6-2. A. Quantification cycle (Cq) values were determined for 40, 15 or 5 ng total RNA isolated from cells derived from the A549 human lung cancer cell-line. The TaqMan™ microRNA RT-PCR assay with ID 1093 from Applied Biosystems® (ABI) or a similar but custom assay for the RNU6-2 nucleolar RNA were used. The two assays were different for only the primers and probes. The linear regression line (ordinary least squares method) for the scatter-plot is also shown. The Pearson correlation coefficient is > 0.99 (P = 0.02). B. An ethidium bromide-stained agarose gel (2%) after electrophoresis of the RT-PCR products for the assays with 40 ng RNA input was transilluminated with ultraviolet light and photographed. Sizes of DNA molecular weight markers (Invitrogen®, Carlsbad, CA) in base-pairs (bp) are shown. The RT-PCR product expected in the custom assay has a size of 75 bp. [file 1756-0500-5-40-S2.TIFF]
